# Mathematical Modeling Suggests That Monocyte Activity May Drive Sex Disparities during Influenza Infection

**DOI:** 10.3390/v16060837

**Published:** 2024-05-24

**Authors:** Tatum S. Liparulo, Jason E. Shoemaker

**Affiliations:** 1 Department of Chemical & Petroleum Engineering, University of Pittsburgh, Pittsburgh, PA 15260, USA; 2 McGowan Institute for Regenerative Medicine, University of Pittsburgh, Pittsburgh, PA 15260, USA; 3 Department of Computational and Systems Biology, University of Pittsburgh, Pittsburgh, PA 15260, USA; 4 Department of Bioengineering, University of Pittsburgh, Pittsburgh, PA 15260, USA

**Keywords:** influenza, sex differences, interferon, monocytes, ODE modeling, systems biology

## Abstract

In humans, females of reproductive age often experience a more severe disease during influenza A virus infection, which may be due to differences in their innate immune response. Sex-specific outcomes to influenza infection have been recapitulated in mice, enabling researchers to study viral and immune dynamics in vivo in order to identify immune mechanisms that are differently regulated between the sexes. This study is based on the hypothesis that sex-specific outcomes emerge due to differences in the rates/speeds that select immune components respond. Using publicly available sex-specific murine data, we utilized dynamic mathematical models of the innate immune response to identify candidate mechanisms that may lead to increased disease severity in female mice. We implemented a large computational screen using the Bayesian information criterion (BIC), wherein the goodness of fit of the competing model scenarios is balanced against complexity (i.e., the number of parameters). Our results suggest that having sex-specific rates for proinflammatory monocyte induction by interferon and monocyte inhibition of virus replication provides the simplest (lowest BIC) explanation for the difference observed in the male and female immune responses. Markov-chain Monte Carlo (MCMC) analysis and global sensitivity analysis of the top performing scenario were performed to provide rigorous estimates of the sex-specific parameter distributions and to provide insight into which parameters most affect innate immune responses. Simulations using the top-performing model suggest that monocyte activity could be a key target to reduce influenza disease severity in females. Overall, our Bayesian statistical and dynamic modeling approach suggests that monocyte activity and induction parameters are sex-specific and may explain sex-differences in influenza disease immune dynamics.

## 1. Introduction

In human infections, females are at increased risk for death and serious illness from outbreak and pandemic strains of influenza A virus (IAV) compared to age-matched males [1]. This trend is most obvious during female reproductive years (18–50 years of age). During the 2009 H1N1 pandemic, females in the United States made up 53.2% of hospitalizations, versus 46.8% for males, and were at higher risk of death than their male counterparts [2,3]. H5N1 infections, while occurring more often in males, result in greater disease severity and mortality for females [4]. During the H7N9 outbreak in 2013–2014, while aged men were the most likely to be hospitalized, females of reproductive age were most likely to die due to infection [5]. Pregnant females experience even greater disease severity during influenza infection than non-pregnant women of reproductive age [6]. These disparities indicate a mechanistic difference in the pathogenesis of IAV between males and females during reproductive years.

Several factors may explain the different infection outcomes between males and females. These can include sex chromosome-linked genes, such as the protein recognition receptor *Tlr7* located on the X chromosome, which is responsible for recognizing viral RNA genomes and has a higher expression in female versus male cells [7], or environmental factors such as nutrition or microbiota [8]. One factor in particular, sex hormone levels, has been shown to have a considerable impact on inflammation, a major driver of respiratory infection disease severity [9]. Estrogen, progesterone, and androgens have receptors on many different innate and adaptive immune cells, such as T cells, dendritic cells (DCs), and natural killer (NK) cells, and the number of receptors for each hormone differs between male and female cells, resulting in different responses to infection between sexes [8]. Estrogens, specifically estradiol (E2), have bipotential effects on immune regulation. At low concentrations, E2 enhances the production of pro-inflammatory cytokines by increasing the activity of Type 1 T helper cells (T_H_1 cells). T_H_1 cells produce interferon-gamma (IFNγ), interleukin-2 (IL-2), and tumor necrosis factor-beta (TNFβ). These cytokines activate macrophages and can increase cell-mediated immunity [10]. Conversely, high doses of E2 reduce the production of these cytokines, dampening macrophage responses [8]. High concentrations of E2 increase the activity of Type 2 T helper cells (T_H_2 cells), which produce anti-inflammatory cytokines (i.e., IL-4 and IL-5) that cause increased antibody protection and eosinophil activation [10]. Females also have greater type I interferon (IFN) activity than males, likely due to E2 promotion of monocytes into inflammatory DCs that produce IFN and other pro-inflammatory cytokines [11,12]. IFNs are responsible for the induction of IFN-stimulated genes, which cause the antiviral responses to be activated in infected and neighboring cells [11]. E2 also promotes the differentiation of bone marrow precursor cells into functional CD11c+ DCs, which increases MCP1/CCL2 synthesis [8]. CCL2 regulates the migration and infiltration of monocytes to the site of infection [13]. Taken together, chromosomal differences and sex hormone differences can differently regulate many aspects of the innate immune response during IAV infection between males and females.

Mathematical modeling is a powerful tool for integrating dynamic, sex-specific immune data into testable frameworks and enabling rigorous, data-driven exploration for potential sex-specific factors that may drive distinct outcomes. Many mathematical models of the immune response to influenza have been developed to answer specific questions about infection kinetics, immune dynamics, and resolution [14,15,16,17]. For example, models have been created to analyze the cause of a double peak in viral loads during infection [18] and why viral loads rebound during bacterial coinfection [19], as well as to determine the effectiveness of therapeutic targets in reducing inflammation during infection [18,20,21,22]. Most of the developed models have used ordinary differential equations (ODEs) to link viral replication and host target cells to cell-to-cell signaling during infection (i.e., interferon and other pro-inflammatory cytokine responses) or immune cell activity. Other types of models, such as agent-based models (ABMs), use a rule-based approach to include spatial and stochastic effects to determine optimal experimental setups [23] and understand spatial effects on infection outcomes [24]. Hernandez-Vargas et al. employed an engineering-based approach to construct a reduced model of viral replication which treats cytokines as inputs and determined that type I interferons caused decreased viral production and dampened immune cell recruitment in aged mice [25]. Using an in silico screen, Ackerman et al. determined that the primary cause of distinct immune responses in high versus low pathogenicity influenza viruses is differences in the rate of interferon production by infected lung cells after infection [14]. These studies provide evidence that mathematical modeling can be an important tool to understand the key drivers of sex differences in the immune response to influenza infection.

Here, we hypothesize that the majority of the immune system is conserved between the sexes and that differences observed between male and female immune outcomes are due to differences in the rates/speeds with which a few immune components respond. Using a previously published model of the influenza-induced innate immune response [14], we created an in silico screen in which an innate immune model is trained on data from male and female mice infected with PR8 H1N1 influenza virus [26] under 13 scenarios. In each scenario, a different set of model parameters are allowed to take on sex-specific parameter values. The Bayesian information criterion (BIC), a criterion used for model selection based on a finite number of models, was used to select the best model scenario. The BIC penalizes large models in favor of smaller models to prevent overfitting. Markov-chain Monte Carlo analysis and global sensitivity analysis of the top performing model were performed to provide rigorous estimates of the sex-specific parameter distributions and to establish the parameter(s) which most effect innate immune responses. Overall, our Bayesian statistical and dynamic model approach suggests that monocyte activity and induction parameters are sex-specific and may explain sex-differences in influenza disease immune dynamics.

## 2. Materials and Methods

### 2.1. Experimental Data Collection from Literature

The data for this paper were gathered from Robinson et al., 2011 [26]. Briefly, male and female mice (n = 15 per sex) were infected with H1N1-PR8 at 100 TCID50. Virus titer, body weight, body temperature, and cytokine/chemokine levels were measured at 1, 3, 5, and 7 days post-infection (dpi). The data were pulled from the figures using WebPlotDigitizer. From these data, we were able to select two of the cytokines to use for our model fitting. The authors do not report IFNα or IFNβ for all four timepoints and across the three experimental conditions; we instead chose IL-6 data to use for fitting, since IL-6 is secreted by infected cells in a similar way to interferon and is a common marker of inflammation [27]. The authors also did not report macrophage concentrations, but did report CCL2, which is a cytokine that recruits macrophages at a known rate [11,13]. Overall, we used viral titer data (log 10 TCID50), IL-6 fold-change data, and CCL2 fold-change data to fit the model.

### 2.2. Model Development

The published model from Ackerman et al. was used [14]. This model was developed as a minimal mechanistic model to retain a high level of confidence in the specific mechanisms within the model while minimizing the number of parameters to be trained. The innate immune model is shown in Equations (1)–(3). The model does not include any cell populations, and instead virus growth is modeled semi-logistically. The virus (*V*) produces interferon (*IFN*) according to mass-action kinetics. Interferon then recruits monocytes (*M*) based on a Hill kinetic term, assuming that a critical concentration of interferon is required to attract macrophages to the site of infection. We modified the original model after determining that a Hill kinetic term would be better suited for modeling virus-specific interferon production. Additionally, the original Ackerman model used a Hill kinetic term to model the interferon induction of macrophage production, which we determined would be better modelled using mass action kinetics.

(1) $$\frac{dV}{dt}=k*V*\left(1-\frac{V}{K}\right)-r_{v,ifn}*IFN*V-r_{v,m}*V*M-d_{v}*V$$

(2) $$\frac{dIFN}{dt}=\frac{k1*V^{n}}{k2+V^{n}}+r_{ifn,m}*M-d_{ifn}*IFN$$

(3) $$\frac{dM}{dt}=r_{m,ifn}*IFN-d_{m}*M$$

### 2.3. Parameter Training and Model Selection

The immune model was simulated in Python using Jupyter Notebook (version 6.1.4, Austin, TX, USA) and using the initial conditions and parameter bounds published in the original papers. The integration was performed using Odeint. The model parameters were determined using scipy.minimize, with a Nelder–Mead optimizer and 100,000 maximum iterations. Log-likelihood (Equation (4)) was used as the cost function for minimization with *i* time points and *j* states. The difference between the value of the data and the model is represented as $y_{i,j}-y_{i,j}^{\prime }$, $\sigma_{i,j}^{2}$ is the standard deviation of each timepoint of the data, and *n* is the overall number of data points.

Table 1 shows the full list of parameters, parameter names, and parameter bounds used. The parameter bounds were taken from [14], with the caveat that the parameter ranges needed to be enlarged to account for differences in the units of the virus, interferon, and monocyte data used in this paper compared to [14].

(4) $$\log likelihood=-\frac{\log\left(2\pi n\right)}{2}-\frac{1}{2}\sum_{i,j}\left[\frac{{\left(y_{i,j}-y_{i,j}^{\prime }\right)}^{2}}{\sigma_{i,j}^{2}}+\log\left(\sigma_{i,j}^{2}n\right)\right]$$

### 2.4. BIC-Guided Model Selection

In this work, we generate multiple model scenarios where we fit a male and female model simultaneously to the experimental data, changing which parameters must be shared between the models at each iteration. The model scenarios were generated using a hypothesis-driven approach, where we specifically excluded parameters such as decay rates that are unlikely to be driving infection and immune response dynamics in the male and female mice. We did not do an exhaustive search of all parameter combinations, due to the large amount of computational time that would be needed to explore 8191 model scenarios, not including analysis of the results. Instead, we focused on relevant combinations of viral- and immune-regulating parameters (13 total scenarios). We use the Bayesian information criterion (BIC) to compare these models (Equation (5)). The BIC is comprised of a log-likelihood term, *L*, and a penalty term for increasing numbers of parameters, such that a larger model with a better log likelihood value will not necessarily result in a lower BIC value. The number of data points is represented by *n*. A lower BIC represents a better model, in that the resultant model has a strong balance between its goodness of fit and the number of free parameters.

(5) $$BIC=k\ln\left(n\right)-2ln(L)$$

### 2.5. Markov-Chain Monte Carlo Parameter Exploration

Markov-chain Monte Carlo (MCMC) analysis was used to further explore the parameter space for the sex-specific model fits using the emcee package. MCMC analysis is useful for systems where a distribution of parameter values satisfies the model, instead of one exact value for each parameter. This is an appropriate method for use in biological systems, given the inherent heterogeneity. The log-prior was set to be a uniform distribution within the parameter bounds. The log-probability, the sum of the log prior and log likelihood, was used for MCMC evaluation. A Metropolis–Hastings algorithm was used to sample the posterior distribution, which uses a weighted random walk [28,29].

The initial amount of virus was allowed to vary between 1 and 2 log10 TCID50/mL and was estimated during fitting. The initial interferon and monocyte values were set to 1 (unitless) since the data were reported in units of fold-change.

The results from using MCMC analysis to explore the parameter space were filtered based on the top 10% of parameter chains, i.e., the chains resulting in the lowest 10% of the -log-likelihood values. This is done to ensure that the algorithm is given enough time to “burn-in” and that we are only analyzing the best-fitting set of parameters. The best 10% of parameter chains were used to identify correlated parameters and compare male and female parameter distributions.

### 2.6. Sensitivity Analysis

A global sensitivity analysis was performed using linear regression to identify the parameter values that most impact model responses. A random parameter space with 1000 parameter sets was generated within ±20% of the best-fit value for the male and female models independently. Then, the model was simulated and the area under the curve (AUC) was calculated for each state. The parameter values and AUC were normalized using the best-fit values for each model. The linear regression module from the scikit-learn package in Python was used to perform a linear regression using the normalized parameter values as the predictors and the normalized AUC values as the output [30].

## 3. Results

### 3.1. Reconstructing Prior Data

**Figure 1:**
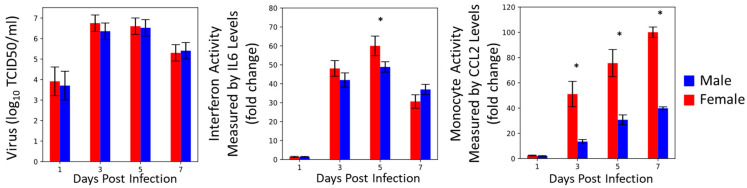
Data used in model fitting that was reconstructed from Robinson et al., 2011 [26]. Female data are shown in red; male data in blue. * indicates significant differences between the sexes at each time point, *p* < 0.05. Please see original publication for detailed experiment protocols.

**Figure 2:**
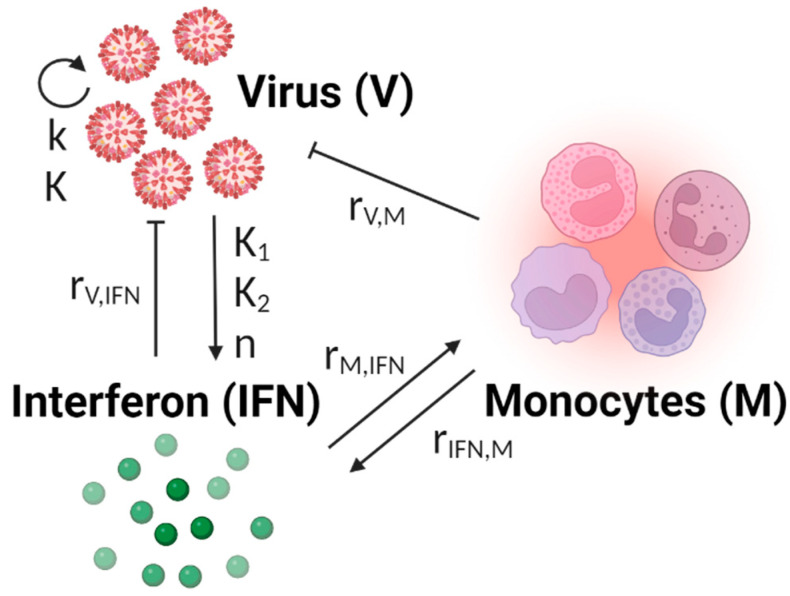
Model diagram of the innate immune model. This model describes the innate immune response to influenza and does not describe the adaptive immune response or infection resolution. Virus growth (V) is modeled using semi-logistic growth and induces interferon (IFN) production. Interferon inhibits viral growth and recruits monocytes (M) to the site of infection. Monocytes also inhibit viral growth and produce additional interferon. The equations describing the model can be found in Section 2.2. The model parameters and the interactions in which they are involved are shown. Created using Biorender.

After extracting the data from Robinson et al., 2011 [26], we reconstructed the data as shown in Figure 1. The viral titers for the male and female mice are not significantly different at 1, 3, 5, or 7 dpi. The interferon activity, measured by the IL-6 fold-change levels, was significantly elevated in female mice at 5 dpi (*p* < 0.05, as reported in the original work) but otherwise not statistically different. The monocyte activity, measured by the CCL2 fold-change levels, was significantly elevated at 3, 5, and 7 dpi in female mice compared to male mice (*p* < 0.05, as reported in the original work). The female mice also had significant increases in morbidity and mortality compared to the male mice ([26]). Given the similarities in viral titers for the male and female mice, it is likely that the significant increase in the proinflammatory immune response in the female mice is a major driver of the reported increase in morbidity and mortality in the female mice. The schematic of the mathematical model of the immune response used to analyze these data is shown in Figure 2.

### 3.2. Innate Immune Mathematical Model Can Fit Immune Response Data from Male and Female Mice

**Figure 3:**
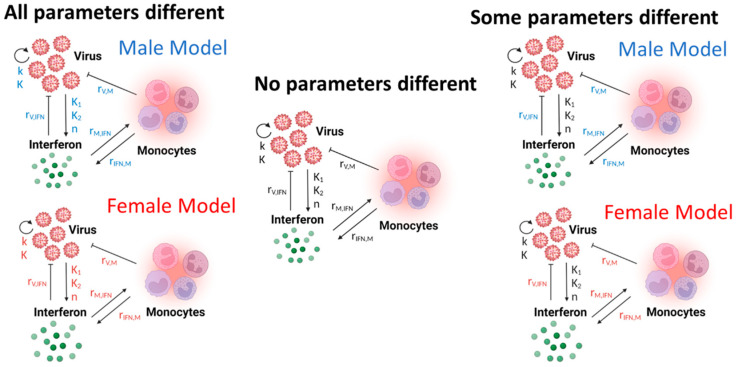
Male and female model scenarios. Example model scenarios considered to determine sex-specific parameters in the innate immune response to influenza. Black parameters indicate shared male and female parameters, while red and blue parameters indicate that the parameters can take unique, sex-specific values. In the All Different model, each parameter can take a unique, sex-specific value. In the All Same model, where no parameters are different, all of the parameters must share values while simultaneously fitting the male and female data. Finally, a pre-selected subset of the parameters can take unique values while the remaining parameters must share a value during simultaneous fitting of the male and female data. The specific parameter subset will be chosen based on prior knowledge of the data and the innate immune behaviors known to be different between males and females. Created using Biorender.

The goal of this work is to use dynamic mathematical modeling to determine differences in immune regulation between male and female mice after H1N1 infection. Differences in immune regulation can be represented by parameters taking on different values within a mathematical model (model shown in Figure 2). In this study, we will propose several scenarios wherein different hypotheses for differences in male and female immune regulation can be evaluated (Figure 3). Each scenario will be evaluated using the BIC, which provides a score that rewards the model for fitting well to the data but penalizes a model for requiring more parameters to fit the data. The lower the BIC, the better the model is for representing the available data.

**Figure 4:**
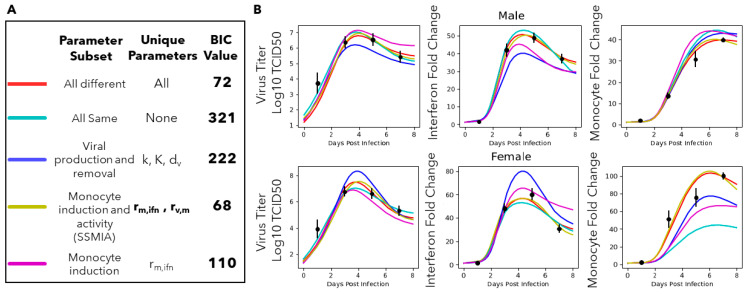
BIC-guided model selection to identify sex-specific parameters. (**A**) Table summarizing a subset of the different model scenarios tested, with model trajectories shown in (**B**). The All Different scenario (red line) resulted in a BIC of 72, which will be considered the baseline BIC that the subsequent models will be compared to. The All Same (light blue line) scenario results in a BIC of 321, which is a significantly worse model fit than the All Different scenario. The viral production and removal scenario (bark blue line) results in a BIC value of 222, again signifying a significantly worse model fit than the All Different scenario. The monocyte induction and activity scenario (yellow line) results in a BIC value of 68, which is a significant improvement over the All Different BIC value, indicating a better model fit and improved model. The monocyte induction scenario (purple line) results in a BIC value of 110, which is a significantly worse model fit than the All Different scenario. The BIC and -log-likelihood values for other tested model scenarios can be found in Supplementary S1. The code to simulate the scenarios shown can be found at https://github.com/ImmuSystems-Lab/Sex-Disparities-Influenza-Infection.git.

The first scenario considered is the All Different scenario, wherein we assume that all of the parameters can differ when comparing the immune responses of infected males and females. In this scenario, the model is trained separately to the male and female data, resulting in a male-specific and a female-specific estimate for each parameter. The model parameters are trained using a constrained optimization algorithm. The parameter bounds are shown in Table 1 and were based on parameter ranges used in [14]. Allowing all the model parameters to take sex-specific values results in a good model fit, indicated by the best-fit line falling within two standard deviations of all data points (red line, Figure 4). This demonstrates that the mechanistic model is capable of fitting to the male and female immunologic data in such a way that the parameters are bounded between physiologically relevant rates. The BIC for this scenario is 72 and this represents a reference number against which future scenarios can be compared.

### 3.3. The Innate Immune Mathematical Model Finds That Male and Female Mice Have Different Rates of Immune Activation to H1N1

The other extreme scenario to consider is that no differences exist between the male and female immune responses. Referring to this scenario as the All Same scenario, we assume that there are no sex differences and, therefore, all the parameters of the immune system model can only take on a single value. The model is trained on the combined male and female data, resulting in a single estimated value for each parameter. Forcing all the parameters to share a value when fitting the male and female models simultaneously results in a poor fit (light blue line, Figure 4B) and a BIC of 321 (Figure 4A). This is significantly larger than the BIC for the All Different scenario, indicating that the All Different scenario provides a better model of the male and female immune response data. At the very least, this allows us to conclude that it is unlikely that the parameters of the immune system model are the same when modeling male and female immune responses.

### 3.4. A Computational Screen of Competing Immune Regulation Scenarios Suggests That Monocyte Induction and Activation Are Potential Sex-Specific Parameters

The All Different and All Same scenarios represent two relatively extreme hypotheses, that the rates of the male and female immune systems are either completely different or completely the same. Here, to robustly determine if a subset of the parameters in the model is sex-specific, BIC-guided model selection was performed to screen 13 scenarios (Section 2.4).

Figure 4 shows a subset of the resulting BIC and trajectories of the best fit achieved for a subset of the scenarios screened. Supplemental Figure S1 shows a graph of the BIC versus -log-likelihood values for all tested scenarios. We first decided to test the hypothesis that viral reproduction could explain differences in disease severity between males and females by allowing *k*, *K*, and *d_v_* to take on sex-specific values. The BIC value for this scenario (viral production and decay) was 222 (Figure 4A), which is significantly larger than the BIC value for the All Different scenario. Looking at the trajectory of this scenario (Figure 4B, dark blue line), we can see that there is a poor model fit to the male and female interferon data and the female monocyte data. Allowing the viral production and decay parameters to be sex-specific resulted in a worse model fit; therefore, it is unlikely that these parameters are sufficient to explain the differences observed in the male and female data. We decided to focus on the monocyte-regulating parameters, since this is the only state with significantly different data between the male and female mice. Allowing just the monocyte induction and activity parameters (*r_m,ifn_* and *r_v,m_*) to take sex-specific values results in a model fit similar to the All Different scenario (yellow and red lines, respectively, Figure 4B) while improving the BIC to 68 (Figure 4A). This corresponds to strong evidence that the model with sex-specific monocyte induction and activity parameters is the better model. Allowing monocyte induction (*r_m,ifn_*) alone to take unique male and female values does not accurately fit the data, particularly with regards to the female interferon and monocyte data (purple line, Figure 4B). The BIC value for this scenario is 110, which is significantly greater than the All Different BIC value, indicating a worse model. Finally, we tested an immune regulating with viral production scenario (*k*, *r_m,ifn_*, *r_v,m_*, *r_ifn,m_*, *r_v,m_*, *r_v,ifn_*, and *K*1), shown in Supplemental Figure S1, that resulted in a BIC value of 70; this is not a significantly improved BIC value compared to the All Different scenario, so we did not further investigate this scenario.

Overall, we have shown that monocyte induction and activity (*r_m,ifn_* and *r_v,m_*) are likely sex-specific parameters leading to the differences we see in infection dynamics between male and female mice, which, from this point on, we will refer to as the “Sex-Specific Monocyte Induction and Activation” scenario, or SSMIA.

### 3.5. Monocyte Induction and Activation Are Differentially Regulated in Male and Female Mice

**Figure 5:**
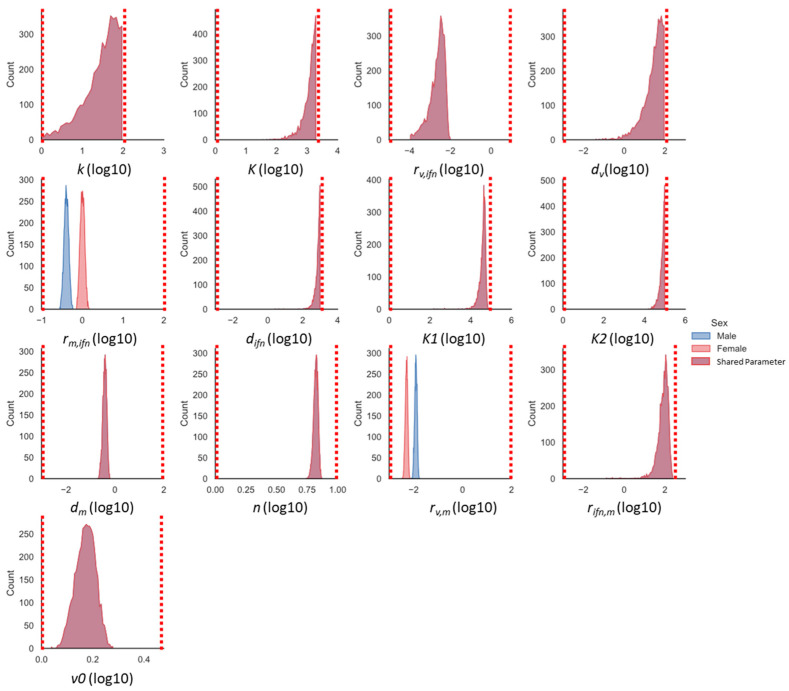
Parameter histograms for SSMIA scenario. Red dotted lines indicate parameter bounds used during MCMC. The purple color indicates parameters shared between the male and female models, while female-specific parameter distributions are shown in red and male-specific parameter distributions are shown in blue.

**Figure 6:**
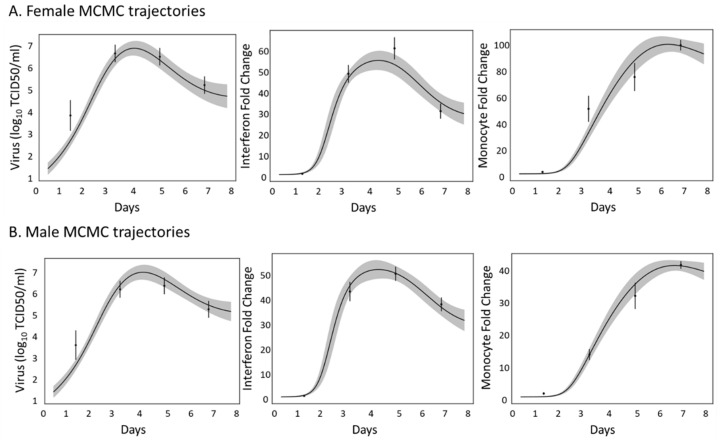
Model trajectories using the top 10% of parameter chains after MCMC analysis of the Best BIC scenario. Top 10% of parameter chains were selected, the parameters were separated into shared, male, or female parameters based on the Best BIC scenario, and Odeint was used to solve the model for each parameter chain. The results were then summarized to show the trajectory of 95% of the model solutions. (**A**) Female trajectories for virus, interferon, and monocyte. All of the trajectories fall within two standard deviations of the data, indicating that MCMC analysis successfully found solutions to the model that fit the data. (**B**) Male trajectories for virus, interferon, and monocyte. All of the trajectories fall within two standard deviations of the data, indicating that MCMC analysis successfully found solutions to the model that fit the data.

The screening method above identifies the scenario wherein the model is most suited for modeling the immune data, but it does not provide a rigorous estimate of the model’s parameter space. MCMC analysis (Section 2.5) was used to estimate the distributions of the parameters for which the model reasonably fit the data. The parameter density distributions for the SSMIA scenario are shown in Figure 5. The x axes are log_10_-scaled, and only the top 10% of the parameter chains are shown to account for burn-in during the initial steps of MCMC analysis and ensure only the parameter chains resulting in the best fit of the model to the data are analyzed (described in Section 2.5). In our SSMIA scenario, we see that the two parameters allowed to take sex-specific values (r_m,ifn_ and r_v,m_) have distinct parameter-density distributions between male and female values, shown by the blue and red distributions, respectively (Figure 5). The dotted red line in the figure indicates bounds used during MCMC simulations. To ensure that MCMC simulations resulted in appropriate model fits, Figure 6 shows the 95% range of model solutions from the MCMC analysis for the male and female models. For all of the states, the model best-fit ranges are within two standard deviations of the data, indicating that the parameter distributions identified by the MCMC analysis do result in a good model fit to the data.

In order to determine if there were differences in parameter distributions between the All Different and Best BIC scenarios, we used MCMC analysis to explore the parameter space of the All Different model (Supplement Figure S2). *K*, *d_ifn_*, *K*1, *K*2, *d_m_*, and *n* have significant parameter overlap between the male and female parameters, indicating that it is unlikely that these parameters are sex-specific. The parameters *r_m,ifn_*, *r_v,m_*, and *r_ifn,m_* have clearly distinct male and female parameter distributions, indicating that these parameters could be sex-specific. We ran a simulation including *r_ifn,m_* in the SSMIA scenario, resulting in a BIC value of 220, which was not a significant improvement over the All Different scenario (not shown); therefore, it is unlikely that *r_ifn,m_* is a sex-specific parameter. The distributions for the male and female *r_v,m_* parameters and male *r_m,ifn_* parameter are similar for the All Different and SSMIA scenarios, while the distribution for the female *r_m,ifn_* scenario has a double-peak that includes larger parameter values in the All Different compared to the SSMIA scenario. The remaining parameters have some overlap between the male and female parameter distributions, but the BIC was not improved when including these parameters in additional model scenarios, which indicates that these parameters are not strongly sex-specific parameters.

**Figure 7:**
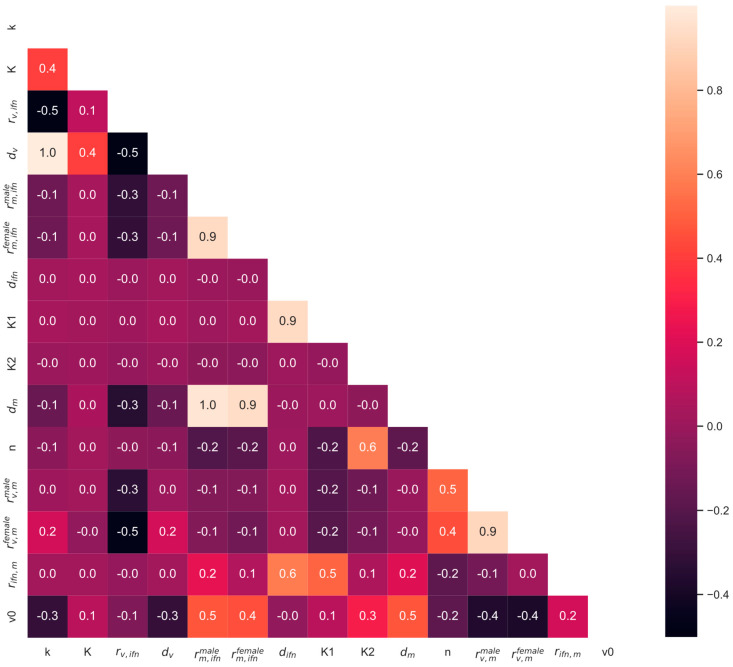
Parameter correlations for the SSMIA scenario. Correlations were determined using the *corr* function from Pandas in Python on the top 10% of parameter chains from MCMC analysis.

Lastly, the parameter chains were analyzed to determine significant correlations between parameters for the SSMIA scenario, shown in Figure 7. While the monocyte induction and activity sex-specific parameter values are not correlated, there are other parameters that are correlated. Specifically, we can see that *d_m_* is perfectly correlated to *r_m,ifn_^male^* and *r_m,ifn_^female^*, and that *d_v_* is perfectly corelated to *k*. Perfect correlations concerning the decay rates of monocytes and the virus indicate that the model could be further simplified according to these correlations to fix the values of the decay parameters by some ratio during fitting, which would further reduce the model complexity.

### 3.6. Global Sensitivity Analysis

**Figure 8:**
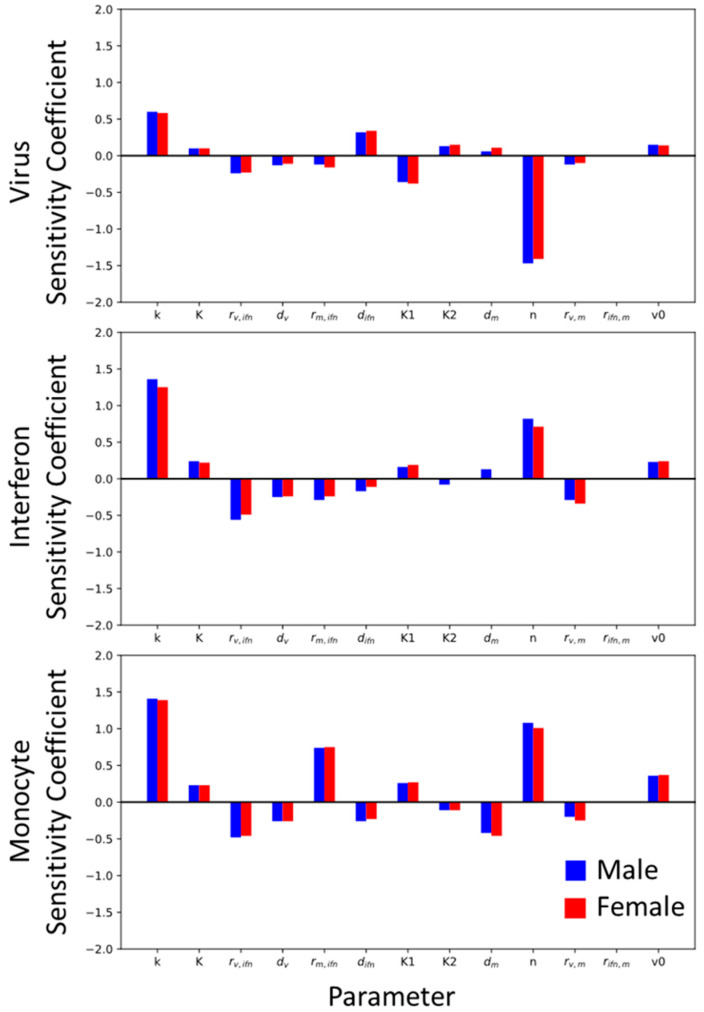
Global sensitivity analysis for the Best BIC model. Sensitivity was calculated using the methods described in Section 2.6. The code used to determine the global sensitivity can be found at https://github.com/ImmuSystems-Lab/Sex-Disparities-Influenza-Infection.git.

We next determined which parameters most affect the immune model’s response and determined how these parameters may differently impact the model output when trained on the male or female data. The male and female global sensitivity results for each state are shown in Figure 8 for the SSMIA scenario. The female and male models are slightly sensitive to viral production and decay (*k* and *d_v_*). The male and female monocyte states are sensitive to *r_m,ifn_*, which was identified as a sex-specific parameter and is one of the two parameters allowed to take unique male and female values. This could support monocyte induction (*r_m,ifn_*) as a sex-specific parameter, since this parameter has a large effect on male and female model outcomes. Additionally, all of the model states for males and females in SSMIA scenarios are sensitive to n, the Hill coefficient. As this is an exponential parameter, it makes sense that small changes would have a large effect on the model outputs. The model is not highly sensitive (sensitivity coefficient < |0.5|) to the remaining parameters, and the male and female models have similar sensitivities to these parameters.

### 3.7. Model Simulations

**Figure 9:**
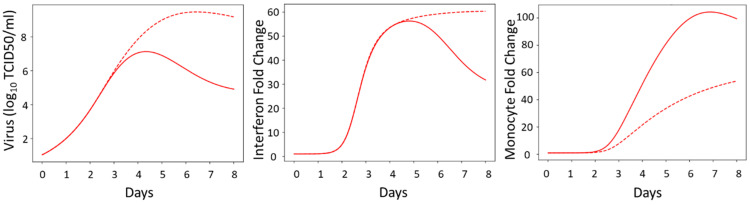
The model predicts that, in the female SSMIA scenario, replacing the female monocyte induction (*r_m,ifn_^female^*) parameter value with the male monocyte induction (*r_m,ifn_^male^*) parameter value, that the decrease in monocyte induction leads to a prolonged infection and excess interferon production. This could indicate a more severe infection. The solid red line shows the nominal female model while the dotted red line shows the nominal female model with the male monocyte induction parameter.

We then wanted to use the model to predict the effectiveness of a treatment strategy that targeted monocyte induction (*r_m,ifn_*) or monocyte activation (*r_v,m_*) in females for severe influenza. Using the SSMIA scenario, we ran two simulations in which each female sex-specific parameter (*r_m,ifn_^female^* or *r_m,ifn_^female^*) was changed to the value of the male-specific parameter in order to determine which pathway would be preferable to target therapeutically. We used parameter values found during the computational screen for the SSMIA scenario (Section 2.3). When changing *r_m,ifn_^female^* to *r_m,ifn_^male^*, keeping all other parameters at their median shared or median female value, we can see, in Figure 9, that the decrease in monocyte induction leads to an increase in viral titers between 3 and 8 dpi, prolonged elevated interferon levels after 4 dpi, and reduced monocyte levels as early as 3 dpi. The elevated virus and interferon levels are suggestive of a more severe infection using *r_m,ifn_^male^*. This suggests that dampening monocyte induction in female mice is not an effective therapeutic approach for improving infection outcomes in females.

**Figure 10:**
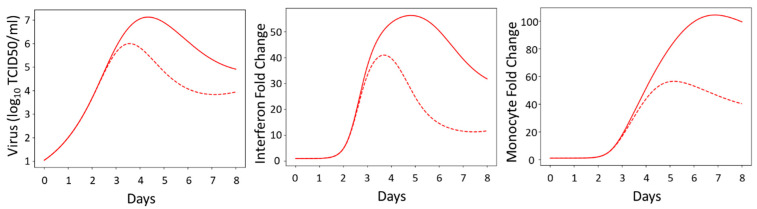
The model predicts that, in the female SSMIA scenario, replacing the female monocyte activity (r_v,m_) parameter with the male monocyte activity (r_v,m_) parameter, that the increase in viral removal capability of the monocyte leads to a decrease in viral titers at 3 dpi and an overall decrease in interferon production. This indicates a shorter, less severe infection. The solid red line shows the nominal female model while the dotted red line shows the nominal female model with the male monocyte activity parameter.

When changing *r_v,m_^female^* to *r_v,m_^male^*, keeping all other parameters at their median shared or median female value, we can see, in Figure 10, that that the increase in the viral removal capability of the monocytes leads to a decrease in viral titers as early as 3 dpi and an overall decrease in interferon production and monocyte recruitment. This suggests a shorter infection duration with less inflammation and less severe disease in the female mice. This result additionally suggests that therapeutics that regulate monocyte differentiation, resulting in increased viral removal rates, could benefit females during influenza infection.

## 4. Discussion

Overall, we were able to use a mathematical model of the innate immune response to analyze sex differences in the immune response to influenza. Our results suggest that allowing the monocyte induction and activity parameters to be sex-specific is sufficient to replicate the trends seen in the male and female murine immune data. Independent, prior studies provide additional support for differences in monocyte induction leading to sex disparities in influenza infection. In females, low levels of circulating estradiol result in increased monocyte recruitment and increased monocyte differentiation into proinflammatory cells [8,10]. This evidence is consistent with our model findings that monocyte induction and activity play a crucial role in severe influenza infection in female mice. Dawson et al. have shown that limiting monocyte infiltration by knocking out CCR2 leads to improved infection outcomes, such as increased survival in mice (sex of mice unclear), despite increases in the viral titer at 5 dpi in those mice [31]. They observed lower monocyte cell counts and increased virus titers at day 5 p.i. These authors did not report any information on interferon in their study. Our simulation results in Figure 9 (Section 3.6) show similar behavior, with lower monocyte counts resulting from a decrease in monocyte induction; however, we are unable to specifically correlate these dynamics to a predicted level of disease severity.

Interestingly, our scenario screen performed in Section 3.4 did not identify interferon activity or induction parameters as being sex-specific. Interferon activity has previously been included in influenza modeling studies and was shown to be a key driver of influenza severity [14,15,16,18]. As shown in Figure 1, the interferon concentration is higher in female mice compared to male mice during infection [26]. The modeling suggests that this difference is driven by differences in the viral load and is not necessarily due to differences in the rate of IFN production. More research is needed to fully understand the role that cytokines and chemokines have in recruiting and polarizing innate immune cells and how differences in immune cell counts and activity differ between males and females, since this additional knowledge could aid model validation. Additionally, our model suggests that hypotheses in the literature concerning viral production as a primary driver of disease severity [32,33] may not be the case when comparing sex differences in disease severity, since the scenario screen in Section 3.4 did not identify viral production as a sex-specific parameter. Host differences in susceptibility to viral production could still contribute to differences in influenza infection outcomes but are likely coupled with differences in the innate immune response based on the results of our study.

While the results in this paper are exciting and represent a novel use of mathematical models in identifying sex differences in influenza infection, important limitations exist, especially with regards to data availability. This makes investigating model behavior difficult; however, our conclusions can still be used to inform future experimental work. Narrowing potential sex-specific effects to monocytes does not show a complete picture of the innate immune response to influenza infection; a model including more innate immune response cell states would greatly benefit analysis and fitting. Neutrophils have been implicated in female responses to influenza infection, and incorporating this state could aid in understanding these sex differences [34]. Additionally, more complete data are needed to clarify the dynamics of the system, specifically within the first few days post-infection. This has been shown to be a key period of IFN and pro-inflammatory cytokine production, leading to differences in disease severity, and differences during this time frame could aid in identifying clear drivers of sex differences during infection [12,35]. Certain model parameters are perfectly correlated, which could be leveraged to reduce the model dimensions and increase the parameter estimation confidence in future modeling studies.

A notable interest for future models will be to incorporate hormones, either as immune cell rate modulators or as a distinct state, to tie hormonal influences on the innate immune response to infection outcomes. For this, we require not only hormone concentrations to be reported, but the identification of menstrual cycle stages for the female mice. Since hormone concentrations change cyclically in female mammals, tracking hormone concentrations alone during influenza infection may not be enough to establish a relationship between estradiol levels in female mice, influenza disease severity, and immune responses. While many murine studies have treated gonadectomized animals with sex hormones to avoid this issue [26], hormone treatment is unlikely to be used for the treatment of severe influenza infections. Rather, we are more interested in determining which immune pathways are most affected by hormones, and then identifying treatment strategies that target those pathways.

The innate immune model only represents the first few days of the immune response, before the adaptive response begins the work of clearing the virus. This model is limited because infections not cleared by the innate immune response would conceivably lead to death of the host, where, biologically, the adaptive response could still lead to viral clearance after a longer period. It is difficult to include the adaptive immune response in models since the innate and adaptive responses occur on very different time scales, and, often, experiments emphasize early time points or late time points for measurements. Models that can successfully identify sex-specific differences across the innate and adaptive immune systems will require greater temporal resolution across the entire infection time period.

## Figures and Tables

**Table 1:** Parameter bounds during model fitting and selection. Parameter bounds were adapted from [14].

Parameter	Lower Bound	Upper Bound	Units	Parameter Name
k	1	100	days^−1^	Maximum viral growth rate
K	1	2000	log_10_(TCID50/mL)	Viral carrying capacity in lungs
r_v,ifn_	1 × 10^−5^	10	days^−1^	Interferon-regulated inhibition of virus replication
d_v_	1 × 10^−3^	500	days^−1^	Nonspecific viral decay rate
r_m,ifn_	1 × 10^−1^	100	days^−1^	Monocyte induction via interferon
d_ifn_	1 × 10^−3^	1000	days^−1^	Nonspecific interferon decay rate
K1	1	100,000	days^−1^	Interferon production from virus
K2	1	100,000	unitless	Apparent dissociation constant
d_m_	1 × 10^−3^	100	days^−1^	Nonspecific monocyte decay rate
N	1	10	unitless	Hill coefficient, activation threshold of virus needed for interferon production
r_v,m_	1 × 10^−3^	100	days^−1^	Monocyte activity on viral removal
r_ifn,m_	1 × 10^−3^	250	days^−1^	Monocyte-regulated interferon production
v0	1	3	log_10_(TCID50/mL)	Initial Virus Titer

## Data Availability

Code for Figure 4 and Figure 8, the BIC and global sensitivity analysis, can be found at https://github.com/ImmuSystems-Lab/Sex-Disparities-Influenza-Infection.git.

## Supplementary Materials

The following supporting information can be downloaded at: https://www.mdpi.com/article/10.3390/v16060837/s1, Figure S1: A plot showing BIC values versus -log-likelihood values for each model scenario considered. Square data points indicate just a single parameter was allowed to be sex-specific, while circles indicate two or more parameters (*or no parameters*) were allowed to be sex-specific. Labels in bold indicate those model scenarios shown in Figure 3. While the majority of the model scenarios shown do not have BIC and -log-likelihood values smaller than the All Different scenario, there is one scenario, Immune regulating with viral production (*k, r_v,ifn_, r_m,ifn_, r_ifn,m_, r_v,m_, K*1), that has a BIC value of 70. This is not a significant improvement in BIC over the All Different scenario, therefore we do not have evidence that this model scenario is better than the All Different scenario; for this reason we did not include this model scenario in Figure 3 or in further analysis; Figure S2: Parameter histograms for All Different scenario. Parameter bounds are the same as used in Figure 4 during MCMC simulations. The female-specific parameter distributions are shown in red and male-specific parameter distributions are shown in blue.
