# Supplementary material for: Mathematical Modeling Suggests That Monocyte Activity May Drive Sex Disparities during Influenza Infection
Source: Viruses. 2024 May 24;16(6):837. doi: 10.3390/v16060837 (PMC11209518; doi:10.3390/v16060837)

Supplementary Materials

Figure S1. A plot showing BIC values versus -log-likelihood values for each model scenario considered. Square data points indicate just a single parameter was allowed to be sex-specific, while circles indicate two or more parameters *(or no parameters)* were allowed to be sex-specific. Labels in bold indicate those model scenarios shown in Figure 3. While the majority of the model scenarios shown do not have BIC and -log-likelihood values smaller than the All Different scenario, there is one scenario, Immune regulating with viral production (k, $r_{v,ifn}$, $r_{m,ifn}$, $r_{ifn,m}$, $r_{v,m}$, $K1$), that has a BIC value of 70. This is not a significant improvement in BIC over the All Different scenario, therefore we do not have evidence that this model scenario is better than the All Different scenario; for this reason we did not include this model scenario in Figure 3 or in further analysis.

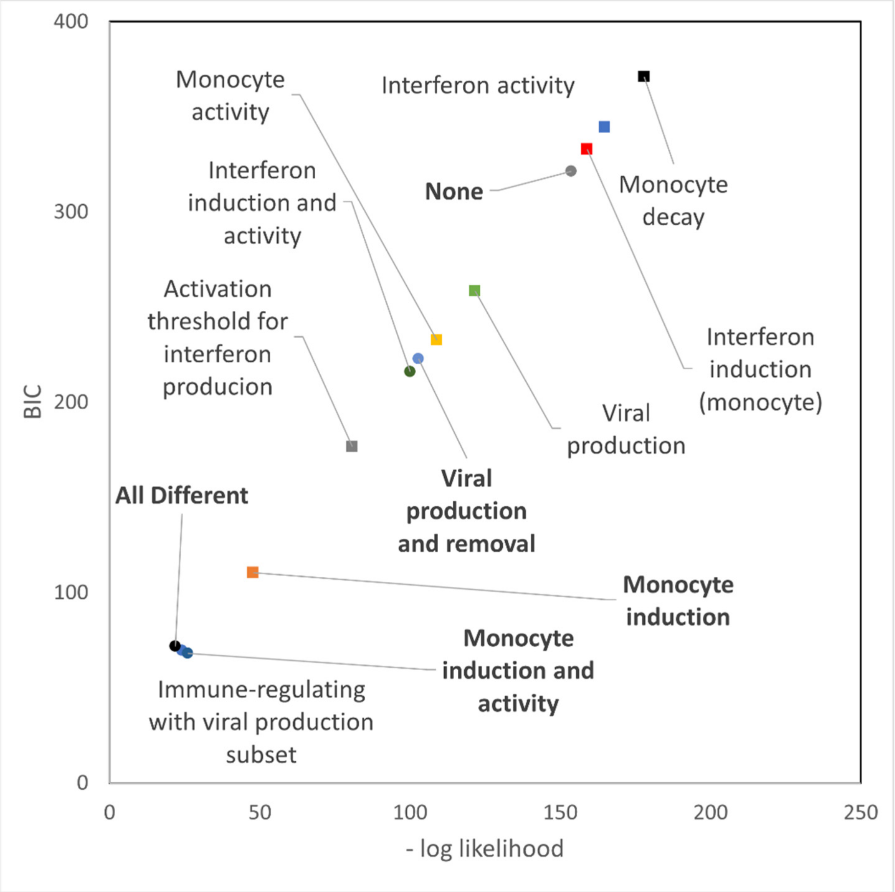

Figure S2. Parameter histograms for All Different scenario. Parameter bounds are the same as used in Figure 4 during MCMC simulations. The female-specific parameter distributions are shown in red and male-specific parameter distributions are shown in blue.

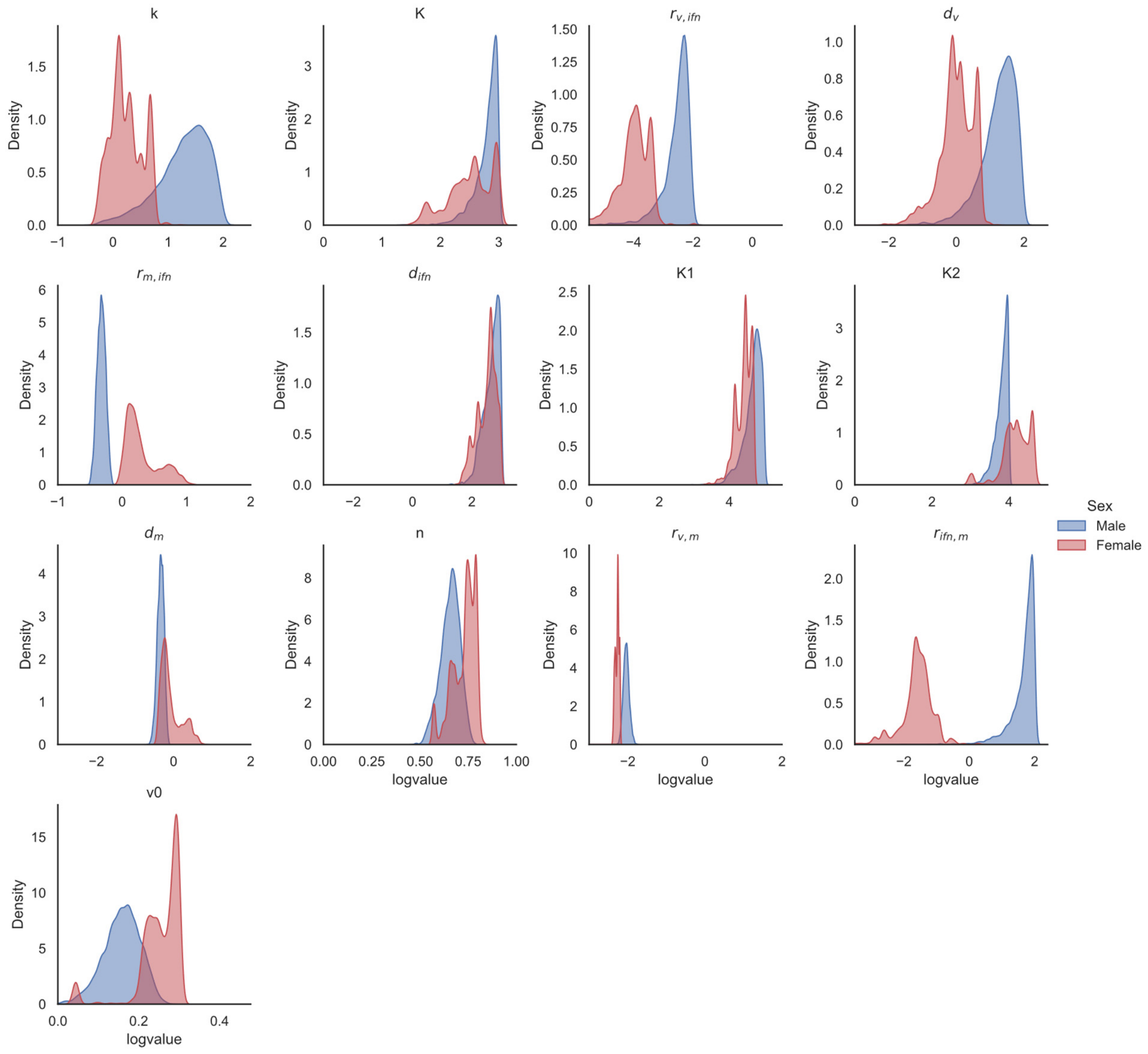

Supplement: Supplementary file 1 [file viruses-16-00837-s001.zip › viruses-2986579-supplementary.pdf]
